# Evaluation of Outcomes Following Hospital-Wide Implementation of a Subcutaneous Insulin Protocol for Diabetic Ketoacidosis

**DOI:** 10.1001/jamanetworkopen.2022.6417

**Published:** 2022-04-07

**Authors:** Priya Rao, Sheng-fang Jiang, Patricia Kipnis, Divyesh M. Patel, Svetlana Katsnelson, Samineh Madani, Vincent X. Liu

**Affiliations:** 1 Kaiser Permanente San Jose Medical Center, San Jose, California; 2 The Permanente Medical Group, Oakland, California; 3 Kaiser Permanente Division of Research, Oakland, California

## Abstract

**Importance:**

Standard diabetic ketoacidosis care in the US includes intravenous insulin treatment in the intensive care unit. Subcutaneous (SQ) insulin could decrease intensive care unit need, but the data are limited.

**Objective:**

To assess outcomes after implementation of an SQ insulin protocol for treating diabetic ketoacidosis.

**Design, Setting, and Participants:**

This cohort study is a retrospective evaluation of a prospectively implemented SQ insulin protocol. The study was conducted at an integrated health care system in Northern California. Participants included hospitalized patients with diabetic ketoacidosis at 21 hospitals between January 1, 2010, and December 31, 2019. The preimplementation phase was 2010 to 2015, and the postimplementation phase was 2017 to 2019. Data analysis was performed from October 2020 to January 2022.

**Exposure:**

An SQ insulin treatment protocol for diabetic ketoacidosis.

**Main Outcomes and Measures:**

Difference-in-differences evaluation of the need for intensive care, mortality, readmission, and length of stay at a single intervention site using an SQ insulin protocol from 2017 onward compared with 20 control hospitals using standard care.

**Results:**

A total of 7989 hospitalizations for diabetic ketoacidosis occurred, with 4739 (59.3%) occurring before and 3250 (40.7%) occurring after implementation. The overall mean (SD) age was 42.3 (17.7) years, with 4137 hospitalizations (51.8%) occurring among female patients. Before implementation, SQ insulin was the first insulin used in 40 intervention (13.4%) and 651 control (14.7%) hospitalizations. After implementation, 98 hospitalizations (80.3%) received SQ insulin first at the intervention site compared with 402 hospitalizations (12.8%) at control sites. The adjusted rate ratio for intensive care unit admission was 0.43 (95% CI, 0.33-0.56) at the intervention sites, a 57% reduction compared with control sites, and was 0.50 (95% CI, 0.25-0.99) for 30-day hospital readmission, a 50% reduction. There were no significant changes in hospital length of stay and rates of death.

**Conclusions and Relevance:**

These findings suggest that a protocol based on SQ insulin for diabetic ketoacidosis treatment was associated with significant decreases in intensive care unit need and readmission, with no evidence of increases in adverse events.

## Key Points

**Question:**

Is a subcutaneous insulin protocol implemented at a hospital level associated with use of intensive care and other outcomes among adults with diabetic ketoacidosis?

**Findings:**

In this cohort study involving 7989 hospitalizations over a 9-year period, implementation of a subcutaneous insulin treatment protocol for diabetic ketoacidosis at a single intervention site, compared with 20 standard care sites, was not associated with increased hypoglycemia but was associated with a relative reduction of direct intensive care unit admission of 57% and a 50% reduction in hospital readmission, with no change in hospital length of stay.

**Meaning:**

These findings suggest that a subcutaneous insulin protocol for treating diabetic ketoacidosis was associated with reduced hospital resource utilization but was not associated with increased hypoglycemia.

## Teaser

This cohort study assesses outcomes after implementation of a subcutaneous insulin protocol for treating diabetic ketoacidosis.

## Introduction

Diabetic ketoacidosis (DKA) is an acute hyperglycemic emergency with the potential for serious morbidity and mortality if not treated promptly. It is a serious complication of diabetes caused by a decrease^1^ in effective circulating insulin and elevations in counter-regulatory hormones, often leading to hyperglycemia, ketone body formation, osmotic diuresis, acid-base and electrolyte derangements, and hypovolemia.^1,2,3,4^ The tenets of management of DKA are the administration of insulin and the correction of the fluid and electrolyte abnormalities. Standard therapy in the US has included continuous intravenous (IV) regular insulin until the resolution of ketoacidosis, with patients typically managed in the intensive care unit (ICU) for close monitoring.^5^ As a result, DKA treatment can be costly in the US, resulting in an estimated annual cost of more than $5 billion.^6^

There is growing interest in DKA treatment that can be achieved safely outside the ICU with non-IV insulin administration. In the United Kingdom, for example, national guidelines recommend the early initiation of fixed rate intravenous insulin infusion^7^ alongside the use of subcutaneous (SQ) insulin. A recent review^5,8^ suggested that there were no substantial advantages or disadvantages of using SQ rapid-acting insulin analogs over IV insulin on the basis of the low quality of the evidence. Some studies^9,10,11,12^ have suggested that SQ short-acting insulin analogs may be as effective as IV insulin. Long-acting insulin glargine was also found to be effective and safe compared with IV regular insulin infusion in a small prospective study.^13^ Despite guideline recommendations for treatment in general medical-surgical wards, ICU treatment of DKA remains common in US health system and hospital settings.^14^

In this study, we examined the outcomes of patients with DKA treated with an SQ insulin protocol including the early use of both rapid-acting and long-acting insulin, in place of IV insulin infusion, followed by rapid-acting insulin injections given over longer periods than previously studied. This SQ DKA protocol was implemented at a single intervention hospital as a quality improvement effort, and we compared outcomes against those from 20 other standard care hospitals in an integrated health care delivery system.

## Methods

This cohort study was approved by the Kaiser Permanente Northern California (KPNC) institutional review board and was determined to be exempt from the need for informed consent according to 45 CFR §46.104(d)(4)(iii). This study follows the Strengthening the Reporting of Observational Studies in Epidemiology (STROBE) reporting guideline.

### SQ Insulin DKA Treatment Protocol

Between January 2010 and January 2016, all 21 KPNC hospitals used a standardized electronic health record (EHR) order set for treating DKA that included IV regular insulin infusion, as well as IV fluids and electrolyte replacement, according to American Diabetes Association guidelines.^15^ ICU admission was standard for all patients treated with IV insulin because hourly glucose checks and insulin titration required a higher level of nursing care than is typically available in other hospital wards. Starting in 2016, specialists at the intervention hospital (KP San Jose Medical Center) designed and implemented a clinical DKA protocol using SQ insulin as primary treatment for all adult (aged ≥18 years), nonpregnant patients with a Glasgow Coma Scale score greater than 8 who did not exhibit non-DKA medical conditions requiring ICU admission. The goal of the protocol was to provide a safe management pathway for patients with DKA outside the ICU while also decreasing nursing workload and improving patient experience.

The protocol was divided into 3 parts: initial emergency department (ED) management, general medical-surgical ward management, and discharge recommendations (Table 1). In the ED, patients meeting DKA protocol criteria were administered weight-based SQ glargine and lispro simultaneously, IV lactated ringer fluid boluses, dextrose 5% with normal saline infusion, and potassium repletion with a primary focus on patients with mild or moderate DKA (Table 1). Patients with severe DKA were also eligible for the SQ insulin protocol if they did not meet the exclusion criteria. DKA severity classifications were determined on the basis of American Diabetes Association criteria (Table 1).

**Table 1.** Overview of Subcutaneous Insulin Protocol Implemented at the Intervention Site for Treatment of Diabetic Ketoacidosis

Criteria	Description
Background	The protocol is divided into initial management at the time of presentation in the ED and post-ED management for medical-surgical units (telemetry units) or observation unit and the discharge recommendations
Inclusion criteria	Adult patients with diabetic ketoacidosis
Exclusion criteria	Age <18 y, pregnancy, any other medical condition that normally would require ICU admission (eg, septic shock, cardiogenic shock, or ST-elevation myocardial infarction), and Glasgow Coma Scale score <8
Notes	The amount of fluid and other aspects of the management must be adjusted for special circumstances, such as heart failure, end-stage kidney disease, and body mass index >40 or overall weight >130 kg^a^
DKA criteria	Severity classification
Glucose, mg/dL	Mild: >250
	Moderate: >250
	Severe: >250
Anion gap, mEq/L	Mild: >10
	Moderate: >12
	Severe: >12
Urine or serum ketone	Mild: positive
	Moderate: positive
	Severe: positive
Arterial pH	Mild: 7.25-7.30
	Moderate: 7.00-7.24
	Severe: <7.00
Serum bicarbonate, mEq/L	Mild: 15-18
	Moderate: 10-15
	Severe: <10
Mental status	Mild: Alert
	Moderate: Alert and drowsy
	Severe: Stupor or coma
Effective serum osmolality	Mild: Variable
	Moderate: Variable
	Severe: Variable
Phase 1	Management in the ED
Standard IV fluids	LR bolus of 2 L, followed by LR at 500 mL/h for a total of 5 L
Dextrose fluids	
If potassium ≤5.5 mEq/L	D5 + 0.45% NaCl + KCl at 40 mEq/L, or 150 mL/h
If potassium >5.5 mEq/L	D5 + 0.45% NaCl without potassium, at 150 mL/h
Long-acting insulin	SQ glargine 0.3 units/kg × 1 or the patient’s usual home dosage of lantus
Rapid-acting insulin	SQ lispro 0.3 units/kg × 1; do not give if glucose is <250 mg/dL; repeat dose in 4 h if glucose is still >250 mg/dL
If glucose ≥250 mg/dL	SQ lispro 0.3 units/kg × 1; repeat dose in 4 h if glucose remains >250 mg/dL; do not give if glucose <250 mg/dL
Glucose monitoring	Monitor glucose every 2 h for 4 h after last dose of high-dose lispro, given then every 4 h
Other	Start 2 peripheral IV lines
Notes	LR and D5 + 0.45% NaCl should be running at the same time
	For patients who had euglycemic ketoacidosis or euglycemic DKA (glucose <250 mg/dL) at presentation, we educated physicians about not administering the high dose of lispro (0.3 units/kg and subsequently 0.2 units/kg) and starting insulin sliding scale after the initial dose of lantus
Phase 2	Management in clinical decision area, medical wards, or telemetry
Standard IV fluids	Continue LR at 500 mL/h for a total of 5 L (if not finished in the ED)
Dextrose fluids	
If potassium ≤5.5 mEq/L	Continue D5 + 0.45% NaCl + KCl at 40 mEq/L for 150 mL/h
If potassium >5.5 mEq/L	Continue D5 + 0.45% NaCl without potassium at 150 mL/h
Long-acting insulin	SQ lantus 0.3 units/kg or patients’ usual dose 24 h after first dose
Rapid-acting insulin	SQ lispro 0.2 units/kg every 4 h until glucose <250 mg/dL; when glucose <250 mg/dL, call physician for insulin sliding scale and discontinue every 4 h lispro
If glucose ≥250 mg/dL	SQ lispro 0.2 units/kg every 4 h until glucose <250 mg/dL
If glucose <250 mg/dL	Call physician for insulin sliding scale and discontinue every 4 h SQ lispro. Start home dose of insulin
Glucose monitoring	Monitor glucose every 2 h for 4 h after last dose of high-dose SQ lispro given; then every 4 h
Electrolyte monitoring	
Potassium	KCl 40 mEq for potassium <4 mEq/L; can give either IV or by mouth
Phosphorus	NaPO_4_ 20 mmol IV for PO_4_ <2 mg/dL
Magnesium	Mg 2 g IV for Mg <2 mg/dL
Diet	Advance diet to diabetic clear diet as tolerated; consider carbohydrate counting for patients with type 1 diabetes
Phase 3	Management at discharge and when DKA is resolved
Type 1 diabetes	
New	Continue insulin glargine at discharge; outpatient endocrinology consultation
Suspected	Continue insulin glargine at discharge; outpatient endocrinology consultation
Known	Discharge on home insulin dose; send EHR message to endocrinologist
Known type 2 diabetes	Discharge on home medications for diabetes and send EHR message to primary care physician and/or referral to chronic conditions program for diabetes

Abbreviations: D5, dextrose 5%; DKA, diabetic ketoacidosis; ED, emergency department; EHR, electronic health record; ICU, intensive care unit; IV, intravenous; KCl, potassium chloride; LR, lactated ringer solution; Mg, magnesium; NaCl, sodium chloride; PO_4_, phosphate; SQ, subcutaneous.

SI conversion factors: To convert anion gap to millimoles per liter, multiply by 1.0; bicarbonate to millimoles per liter, multiply by 1.0; glucose to millimoles per liter, multiply by 0.0555; magnesium to millimoles per liter, multiply by 0.4114; potassium to millimoles per liter, multiply by 1.0; and phosphorus to millimoles per liter, multiply by 0.323.

^a^ Body mass index is calculated as weight in kilograms divided by height in meters squared.

Post-ED management in general medical-surgical wards focused on ongoing volume expansion with lactated ringer fluid and weight-based lispro every 4 hours until the blood glucose level was less than or equal to 250 mg/dL (to convert to millimoles per liter, multiply by 0.0555). Dextrose 5% with normal saline infusion and potassium chloride were continued until patients were started on sliding scale insulin protocol (ie, a variable SQ insulin dose dependent on capillary blood glucose measurements). Electrolytes were monitored every 4 hours and repleted as needed. At the time of discharge, an outpatient endocrinology consultation was requested for all patients with new-onset type 1 diabetes. For patients with established endocrinology clinicians, an EHR message was sent for postdischarge follow-up, and patients with type 2 diabetes were referred to chronic conditions program for diabetes monitoring and follow-up. Besides the intervention site, other KPNC facilities continued to provide standard DKA care with the existing IV insulin EHR order set, except for a single additional facility that implemented the protocol on January 1, 2019.

### Preimplementation and Postimplementation Periods

To evaluate the outcomes associated with the SQ DKA protocol implementation, we defined the preimplementation period as January 1, 2010, to December 31, 2015, and the postimplementation period as January 1, 2017, to December 31, 2019, and compared the outcomes between the periods at the intervention site vs the remaining KPNC facilities using a difference-in-differences approach. Our primary exposure of interest was the period of DKA treatment (preimplementation vs postimplementation). We excluded 2016 data from analysis because the protocol was not yet fully implemented at the intervention site. We also removed data from 2019 forward from the 1 additional facility that began SQ DKA protocol implementation on January 1, 2019.

### DKA Cohort and Characteristics

We evaluated characteristics, treatments, and outcomes among adult inpatients treated for DKA identified by principal hospital *International Classification of Diseases, Ninth Revision *and *International Statistical Classification of Diseases and Related Health Problems, Tenth Revision *diagnosis codes (250.10, 250.11, 250.12, 250.13, E11.10, E11.11, E10.10, E10.11, E13.10, and E13.11) using existing EHR methods.^16,17,18,19,20^ We also required patients to have evidence of ketosis based on a positive finding of ketones in serum or urine samples. Patient demographic characteristics included age, gender, body mass index (weight in kilograms divided by height in meters squared) at the time of admission, race and ethnicity (Asian, Black, Hispanic, White, and any other race or ethnicity, including unknown race or ethnicity), and neighborhood deprivation index.^21^ Race and ethnicity were assessed in this study because of differences in the prevalence and outcomes of diabetes across racial and ethnic groups. Hospitalization characteristics included presentation through the ED and acute and chronic severity of illness metrics, including the Comorbidity Point Score version 2, which is a scalar measure of comorbid disease burden based on a 1-year look back at diagnoses, and the Laboratory and Acute Physiology Score version 2, which is based on laboratory values, vital signs, and neurological status at the time of hospital admission.^16,17,18,19,20^ We identified common DKA laboratory values, including the first serum values for tests such as glucose, anion gap, blood urea nitrogen, bicarbonate, sodium, white blood cell count, creatinine, pH, and potassium. We excluded 23 patients from analysis who did not have any glucose values documented. The rates of missing data were low for all variables except pH values (eTable in the Supplement).

### DKA Treatment, Process Measures, and Outcomes

We identified all insulin treatments administered during hospitalization using the EHR medication administration record and focused on the first dose administered, as well as the total and weight-adjusted insulin dosage units of any type within the first 12, 24, and 48 hours of hospitalization. We identified insulin administration routes as either IV or SQ. We identified IV fluids administered to patients, including 5% dextrose, 10% dextrose, normal saline, or lactated ringer solution. We quantified the time from hospital arrival to the first glucose value less than 250 mg/dL and to anion gap closure (based on a value <16 mEq/L [to convert to millimoles per liter, multiply by 1.0], the upper limit of normal in the KPNC laboratory), which are measures frequently used to determine the response to the treatment of DKA. Finally, we assessed the incidence of hypoglycemia, defined as any serum glucose value less than 70 mg/dL, over the entire hospitalization. We also evaluated the frequency of ICU admission either directly from the ICU or later during the DKA hospitalization. Our outcomes of interest included mortality within 30 days of hospital admission, hospital readmission within 30 days of discharge, and overall hospital length of stay.

### Statistical Analysis

We reported data as number (percentage), mean (SD), or median (IQR) and conducted all analyses using SAS statistical software version 9.4 (SAS Institute). For unadjusted preimplementation and postimplementation comparisons, we used 2-sided *t*, Wilcoxon rank-sum, χ^2^, or Fisher exact tests. *P* < .05 denoted significance. Data analysis was performed from October 2020 to January 2022.

To compare the preimplementation vs postimplementation change in outcomes at the intervention site vs other sites using standard care, we used generalized estimating equations models to estimate the intervention effect and account for within-patient correlations for patients with multiple DKA encounters. The intervention effect was obtained from an interaction between treatment and period effects in the models (log difference-in-differences). The models controlled for fixed hospital effects, age, sex, race, body mass index, Comorbidity Point Score version 2, Laboratory and Acute Physiology Score version 2, and first serum values of glucose, anion gap, and bicarbonate. All continuous covariates entered the model linearly except for body mass index, which was categorized into 5 groups (underweight, reference, overweight, obese, and missing). Binary outcomes were modeled using a binomial distribution with a logit link, and continuous outcomes used a γ distribution with a log link.

## Results

Our study included a total of 7989 DKA hospitalizations occurring among 5046 patients, including 4739 hospitalizations (59.3%) occurring during the preimplementation period and 3250 (40.7%) occurring during the postimplementation period (Table 2). The overall mean (SD) age of the hospitalization cohort was 42.3 (17.7) years, and 4137 encounters (51.8%) included female patients. DKA hospitalizations at the intervention site represented 5.3% (420 hospitalizations) of the entire sample, with unique patients at the intervention site representing 5.2% (260 patients) of all patients. The mean (SD) body mass index of the cohort was 26.2 (7.4), with most patients in the reference weight category.

**Table 2.** Baseline Characteristics and Selected Laboratory Values of Patients Treated During DKA Hospital Encounters at the Intervention Site and Other Regional Sites During the Preimplementation and Postimplementation Periods

Variable	Value, mean (SD)			
	Intervention site		Standard care sites	
	Preimplementation	Postimplementation	Preimplementation	Postimplementation
Hospital encounters, No.	298	122	4441	3128
Unique patients, No.	173	87	2703	2083
Age, y	37.5 (15.5)	39.6 (16.9)	42.2 (17.6)	43.0 (17.8)
Sex, No. (%), hospital encounters				
Female	166 (55.7)	57 (46.7)	2345 (52.8)	1569 (50.2)
Male	132 (44.3)	65 (53.3)	2096 (47.2)	1559 (49.8)
Body mass index, No. (%), hospital encounters				
Underweight	30 (10.1)	25 (20.5)	674 (15.2)	560 (17.9)
Reference	183 (61.4)	64 (52.5)	2752 (62.0)	1779 (56.9)
Overweight	40 (13.4)	23 (18.9)	523 (11.8)	405 (12.9)
Obese	44 (14.8)	9 (7.3)	459 (10.3)	353 (11.3)
Missing	1 (0.3)	1 (0.8)	33 (0.7)	31 (1.0)
Race or ethnicity, No. (%), hospital encounters				
Asian	8 (2.7)	4 (3.3)	189 (4.3)	179 (5.7)
Black	21 (7.1)	19 (15.6)	952 (21.4)	692 (22.1)
Hispanic	122 (40.9)	45 (36.9)	813 (18.3)	626 (20.0)
White	122 (40.9)	47 (38.5)	2196 (49.5)	1461 (46.7)
Other^a^	35 (8.4)	7 (5.7)	291 (6.5)	170 (5.5)
Neighborhood Deprivation Index	−0.30 (0.8)	−0.34 (0.7)	0.14 (1.0)	0.23 (1.0)
Comorbidity Point Score version 2 score (comorbidity burden)	26.8 (29.3)	30.9 (30.4)	33.3 (37.9)	37.5 (46.9)
Laboratory and Acute Physiology Score version 2 (acute severity of illness)	85.7 (34.8)	85.8 (41.3)	82.9 (35.6)	88.9 (38.2)
Visit started in emergency department, No. (%), hospital encounters	292 (98.0)	120 (98.4)	4335 (97.6)	3085 (98.6)
Emergency department length of stay, h	5.4 (2.7)	6.5 (3.5)	5.6 (3.6)	6.7 (6.4)
Elapsed length of stay at admit order, h	3.8 (1.8)	3.7 (1.4)	4.1 (2.5)	3.9 (4.7)
Diabetes history before admission, No. (%), hospital encounters				
Any prior diagnosis of diabetes	269 (90.3)	112 (91.8)	4159 (93.7)	2798 (89.5)
Prior type 1 diabetes diagnosis	184 (61.7)	74 (60.7)	2509 (56.5)	1529 (48.9)
Prior type 2 diabetes diagnosis	85 (28.5)	38 (31.2)	1650 (37.2)	1269 (40.6)
Most recent hemoglobin A_1C_, %	10.7 (2.5)	11.4 (2.6)	11.0 (2.5)	11.5 (2.5)
Outpatient insulin use before DKA, No. (%), hospital encounters	173 (58.1)	75 (61.5)	2621 (59.0)	1756 (56.1)
First serum laboratory value				
Glucose, mg/dL	510.0 (218.8)	532.2 (240.1)	544.5 (233.6)	575.5 (260.2)
Anion gap, mEq/L	23.1 (6.4)	25.4 (6.5)	23.7 (6.8)	25.0 (6.7)
Blood urea nitrogen, mg/dL	22.4 (14.0)	23.7 (18.7)	24.5 (16.7)	27.3 (18.6)
Bicarbonate, mEq/L	12.8 (5.8)	11.9 (6.3)	13.1 (6.0)	12.8 (6.1)
Sodium, mEq/L	133.7 (5.7)	131.2 (5.4)	135.0 (6.3)	131.8 (6.2)
White blood cell count, cells/μL	13 900 (6 900)	14 600 (7 600)	13 100 (6 400)	13 600 (6 700)
Creatinine, mg/dL	1.1 (0.8)	1.3 (0.5)	1.3 (1.1)	1.5 (1.1)
Potassium, mEq/L	4.8 (0.8)	4.8 (0.9)	5.0 (0.9)	4.7 (0.9)
Chloride, mEq/L	96.9 (7.2)	93.8 (6.9)	96.9 (7.4)	94.0 (7.3)
Maximum chloride serum value in 24 h, mEq/L	108.7 (5.6)	106.3 (4.5)	109.7 (6.2)	108.5 (6.1)
Arterial pH	7.2 (0.1)	7.1 (0.2)	7.3 (0.1)	7.2 (0.1)
Venous pH	7.2 (0.2)	7.2 (0.2)	7.2 (0.1)	7.1 (0.1)
Initial serum glucose value <250 mg/dL, No. (%), hospital encounters	26 (8.7)	7 (5.7)	296 (6.7)	152 (4.9)
Total count of glucose values measured	24.3 (14.4)	18.6 (10.1)	29.1 (20.3)	24.2 (16.2)
Severity of DKA, No. (%), hospital encounters				
Mild	8 (2.7)	3 (2.5)	256 (5.8)	63 (2.0)
Moderate	195 (65.4)	62 (50.8)	2746 (61.8)	1963 (62.8)
Severe	95 (31.9)	57 (46.7)	1439 (32.4)	1102 (35.2)

Abbreviation: DKA, diabetic ketoacidosis.

SI conversion factors: To convert anion gap to millimoles per liter, multiply by 1.0; bicarbonate to millimoles per liter, multiply by 1.0; blood urea nitrogen to millimoles per liter, multiply by 0.357; chloride to millimoles per liter, multiply by 1.0; creatinine to micromoles per liter, multiply by 88.4; glucose to millimoles per liter, multiply by 0.0555; hemoglobin A_1C _to proportion of total hemoglobin, multiply by 0.01; potassium to millimoles per liter, multiply by 1.0; sodium to millimoles per liter, multiply by 1.0; and white blood cell count to cells ×10^9^/L, multiply by 0.001.

^a^ Refers to any race or ethnicity not reported as Asian, Black, White, or Hispanic, including unknown race or ethnicity.

Nearly all DKA hospitalizations started in the ED at both intervention (412 hospitalizations [98.1%]) and standard care (7420 hospitalizations [98.0%]) sites (Table 2). At the intervention site, the mean (SD) first serum laboratory glucose values were similar before (510.0 [218.8] mg/dL) and after (532.2 [240.4] mg/dL) implementation. Initial anion gap values were higher in the postimplementation period compared with the preimplementation period (25.4 [6.5] vs 23.1 [6.4] mEq/L), as were serum creatinine values (1.3 [0.5] mg/dL in the postimplementation period vs 1.1 [0.8] mg/dL in the preimplementation period [to convert to micromoles per liter, multiply by 88.4]). At the standard care sites, glucose, anion gap, and creatinine were higher in the postimplementation period compared with the preimplementation period.

In the preimplementation period, SQ insulin was the first insulin administered in 40 intervention site hospitalizations (13.4%) and 651 standard care site hospitalizations (14.7%) (Table 3). In the postimplementation period, 98 intervention site hospitalizations (80.3%) and 402 standard care site hospitalizations (12.8%) received SQ insulin as their first insulin treatment. Over the first 48 hours of hospitalization, the total numbers of insulin units administered at the intervention site did not differ between the preimplementation and postimplementation periods (122.5 [70.9] vs 126.2 [75.4] units), whereas the total numbers of insulin units increased at the standard care sites (109.6 [68.3] units before implementation vs 121.9 [74.0] units after implementation).

**Table 3.** Treatments and Process Measures for Patients With Diabetic Ketoacidosis During the Preimplementation and Postimplementation Phases at the Intervention Site Compared With Other Regional Sites

Variable	Hospital encounters, No. (%)			
	Intervention site		Standard care sites	
	Preimplementation	Postimplementation	Preimplementation	Postimplementation
Hospital encounters, No.	298	122	4441	3128
Unique patients, No.	173	87	2703	2083
First insulin treatment route				
Intravenous	258 (86.6)	24 (19.7)	3790 (85.3)	2726 (87.2)
Subcutaneous	40 (13.4)	98 (80.3)	651 (14.7)	402 (12.8)
Insulin dosage, mean (SD), units				
Total within 12 h	41.7 (28.0)	60.4 (34.7)	39.0 (27.9)	45.4 (25.9)
Total within 24 h	74.6 (42.0)	89.2 (53.1)	68.9 (45.3)	80.1 (45.2)
Total within 48 h	122.5 (70.9)	126.2 (75.4)	109.6 (68.3)	121.9 (74.0)
Weight-based insulin dosage, mean (SD), units				
Total within 12 h	0.6 (0.4)	0.8 (0.6)	0.5 (0.4)	0.6 (0.4)
Total within 24 h	1.0 (0.5)	1.2 (0.8)	0.9 (0.6)	1.1 (0.6)
Total within 48 h	1.6 (0.9)	1.7 (1.1)	1.5 (0.8)	1.6 (0.9)
Received fluid type during hospitalization				
5% dextrose	253 (84.9)	106 (86.9)	4016 (90.4)	2510 (80.2)
10% dextrose	9 (3.0)	1 (0.8)	80 (1.8)	48 (1.5)
Normal saline	298 (100.0)	106 (85.2)	4436 (99.9)	3115 (99.6)
Lactated ringer solution	68 (22.8)	113 (96.6)	480 (10.8)	1187 (38.0)
Received oral hypoglycemic	27 (9.1)	13 (10.7)	414 (9.3)	305 (9.8)
Received vasopressor agent	10 (3.4)	1 (0.8)	57 (1.3)	55 (1.8)
Received endocrinology visit within 2 wk of discharge	21 (7.1)	15 (12.3)	563 (12.7)	403 (12.9)
Required 50% dextrose treatment	22 (7.4)	9 (7.4)	483 (10.9)	345 (11.0)
Serum glucose <70 mg/dL at any point	36 (12.1)	11 (9.0)	699 (15.7)	281 (9.0)
Time to glucose <250 mg/dL, mean (SD), h	9.5 (6.8)	11.3 (6.8)	9.0 (7.2)	10.7 (8.0)
Glucose rebound >250 mg/dL	89 (29.9)	25 (20.5)	1078 (24.3)	798 (25.5)
Time to anion gap <16 mEq/L, mean (SD), h	9.4 (6.2)	9.4 (4.7)	9.5 (6.3)	10.1 (6.1)
Admission to intensive care unit				
Direct	202 (67.8)	34 (27.9)	3357 (75.6)	2488 (79.5)
Late	6 (2.0)	4 (3.3)	64 (1.4)	45 (1.4)

SI conversion factors: To convert anion gap to millimoles per liter, multiply by 1.0; glucose to millimoles per liter, multiply by 0.0555.

At both the intervention and standard care sites, the number of hours until the first documentation of a serum glucose level less than 250 mg/dL increased modestly in the postimplementation vs preimplementation periods (Table 3). The number of hours until the first documented anion gap less than 16 mEq/L was similar at the intervention site and longer at standard care sites. Overall, there was no significant difference in the use of 50% dextrose for treating hypoglycemia in the intervention site, whereas the proportion of patients having evidence of hypoglycemia based on any glucose value less than 70 mg/dL decreased. After hospital discharge, the proportion of hospitalizations having an endocrinology visit within 2 weeks of discharge increased at the intervention site from 7.1% (21 hospitalizations) to 12.3% (15 hospitalizations), whereas there was no change at standard care sites (563 hospitalizations [12.7%] vs 403 hospitalizations [12.9%]).

At the intervention site, there was a substantial decrease in the proportion of hospitalizations directly admitted to the ICU, from 67.8% (202 hospitalizations) in the preimplementation period to 27.9% (34 hospitalizations) in the postimplementation period (χ^2^_1_ = 56.027; *P* < .001), with no change in the proportion of hospitalizations requiring a later admission to the ICU (*P* = .49, Fisher exact test) (Table 3). At standard care sites, direct ICU admission increased from 75.6% (3357 hospitalizations) to 79.5% (2488 hospitalizations) (χ^2^_1_ = 16.268; *P* < .001). After implementation, 30-day readmissions decreased at the intervention site from 21.1% (63 hospitalizations) before implementation to 9.8% (12 hospitalizations) after implementation, whereas they were unchanged at the standard care sites (789 hospitalizations [17.8%] before implementation vs 547 hospitalizations [17.5%] after implementation) (Table 4). Mortality within 30 days of DKA hospitalization was not significantly different at the intervention site (1 patient in the postimplementation period) or standard care sites (48 hospitalizations [1.1%] before vs 35 hospitalizations [1.1%] after implementation), whereas the length of stay decreased modestly in both groups after implementation.

**Table 4.** Unadjusted Outcome Measures Following Implementation at the Intervention Site vs Regional Comparators

Outcome	Patients, No. (%)					
	Intervention site			Standard care sites		
	Preimplementation	Postimplementation	*P* value	Preimplementation	Postimplementation	*P* value
Mortality within 30 d	0	1 (0.8)	.29^a^	48 (1.1)	35 (1.1)	.88^b^
Readmission within 30 d	63 (21.1)	12 (9.8)	.006^b^	789 (17.8)	547 (17.5)	.75^b^
Overall hospital length of stay, mean (SD), h	64.6 (68.6)	56.2 (64.3)	.25^c^	62.5 (72.3)	58.5 (56.6)	<.001^c^

^a^ Calculated with Fisher exact test.

^b^ Calculated with χ^2^ test.

^c^ Calculated with *t* test.

Using difference-in-differences regression (Table 5), the adjusted rate ratio for ICU admission was lower at the intervention site compared with the standard care sites, at 0.43 (95% CI, 0.33-0.56), a 57% reduction, whereas the adjusted rate ratio for 30-day readmission was also lower at 0.50 (95% CI, 0.25-0.99), a 50% reduction. The adjusted rate ratio for hospital length of stay was not significant (0.97; 95% CI, 0.76-1.23). Because only a single death occurred in the intervention site in each of the preimplementation and postimplementation periods, the adjusted rate ratio for mortality could not be estimated reliably.

**Table 5.** Unadjusted and Adjusted Rate Ratios of Outcomes at the Intervention Site and Standard Care Sites Before and After Implementation

Outcome	Rate ratio (95% CI)	
	Unadjusted	Adjusted
Intensive care unit admission during DKA	0.44 (0.33-0.59)	0.43 (0.33-0.56)
Readmitted within 30 d of DKA	0.47 (0.22-0.99)	0.50 (0.25-0.99)
Hospital length of stay	0.90 (0.70-1.15)	0.97 (0.76-1.23)

Abbreviation: DKA, diabetic ketoacidosis.

## Discussion

In this cohort study, we examined the outcomes after implementation of an SQ insulin treatment protocol for DKA at a single intervention site compared with 20 standard care sites over a 9-year period. After implementation, the use of SQ insulin as the first treatment in DKA increased from 13.4% to 80.3% at the intervention site while it remained unchanged at standard care sites. This change in practice had no impact on the mean total insulin dosage received by patients with DKA within their first 48 hours of treatment. Overall, the protocol was associated with a 57% relative decrease in ICU admissions at intervention sites and a 50% relative decrease in 30-day readmissions compared with standard care sites. The use of an SQ insulin-driven protocol appeared to be safe, with no associated increases in the incidence of hypoglycemic events during hospitalization or 30-day mortality.

DKA is the most common acute hyperglycemic emergency among people with diabetes and is associated with significant morbidity and health care cost.^1,4^ Hospitalizations for DKA are also resource-intensive in the US, requiring ICU admission and reportedly costing as much as $26 566 per admission.^22,23^ In the United Kingdom, where ICU utilization for DKA may be lower, the cost of DKA hospitalizations has been reported to be lower.^24^ The ICU is a scarce resource that can become critically strained during seasonal influenza surge or as evidenced by the COVID-19 pandemic.^25^ Thus, many US critical care specialists have routinely identified DKA as a diagnosis well-suited for treatment in non-ICU settings if the right patients can be identified and appropriate treatment and monitoring can be put in place.^26,27^ Despite guidelines recommending non-ICU treatment of DKA, evidence from the general population in the US confirms that many patients are treated in ICU settings.^14^

Although SQ regular insulin has not been accepted as a therapy for DKA given its pharmacokinetics^28^ and slower attainment of effective plasma insulin concentrations,^29^ rapid-acting SQ insulin lispro has a substantially earlier and greater peak serum insulin concentration.^30,31^ Prior studies^32,33,34^ have reported large IV insulin doses in DKA treatment, with 1 study showing an average administration of 216 units within the first 3 hours of treatment. Our protocol included a dose of long-acting insulin glargine administered up front along with SQ insulin lispro at every 4 hours, which is an approach used in United Kingdom DKA guidelines.^7^ We found that the protocol was effective with a similar time needed to reach a glucose less than 250 mg/dL and, more importantly, to anion gap closure without evidence of increased adverse outcomes. More recently, another study^7^ has found that short-acting insulin given SQ up to every 2 hours was safe and effective in treatment of DKA.

Given that the insulin is administered SQ, factors that affect absorption into the bloodstream, including obesity, the use of vasoconstricting drugs, and blood pressure, are important to consider in identifying the right patients for SQ insulin treatment.^35^ Typically, 100-unit insulin volumes of no greater than 0.5 mL are injected into a discrete area because a larger volume of insulin may impede absorption.^36^ Although the protocol used in this study did not have a specific weight limitation, we have recently revised our protocol to exclude patients whose weight is 166 kg or more out of concerns related to insulin absorption.

### Strengths and Limitations

The primary strength of this study was that we were able to examine relative changes resulting from protocol implementation at the intervention site against a large and diverse set of comparator sites. This approach allowed us to address challenges commonly seen with before-and-after studies, which fail to account for ongoing secular changes in practice. In this study, we found that practice at the standard care sites remained largely unchanged in the postimplementation period, suggesting that our findings were the result of changes in practice unique to the intervention site. We were also able to evaluate posthospital outcomes, because patients with DKA are frequently rehospitalized over time, and we found a reduction in readmission rates at the intervention site. Our integrated health care delivery system and comprehensive single EHR system facilitated the identification and tracking of patients after DKA; thus, the protocol’s default consultation for endocrinology follow-up and population management among patients with newly diagnosed diabetes may have played a role in reducing readmissions.

There are several limitations that should be noted. First, our study included patients with DKA based on principal diagnosis codes and, thus, represented a subset of all patients who might have received treatment for DKA. Also, although we used clinical EHR data to establish DKA and its severity, it is possible that patients may have been misclassified. Second, our study was conducted within a single regional integrated health care delivery system that incorporates numerous regional care protocols, which may differ from those in place in other hospital and health systems. Thus, additional study will be needed to assess the generalizability of this protocol, particularly in locales where SQ insulin use in DKA is more common. Third, although we did identify an association with the intervention, this was a retrospective evaluation of a prospectively implemented protocol arising from a quality improvement effort; thus, the results should be confirmed with prospective studies. Fourth, the endocrine outpatient follow-up used in the protocol was directed toward patients with type 1 diabetes, and care for patients with type 2 diabetes could still be improved after DKA with careful care coordination. Fifth, we did not examine all adjunctive treatments given to patients with DKA (eg, fluid type and volume, and bicarbonate or potassium treatment), which could impact outcomes. Sixth, a minority of patients at standard care sites before implementation underwent initial SQ insulin treatment, which likely reflects ongoing variability in DKA treatment patterns. Seventh, although we did adjust for patient-level factors, we did not examine variability in practice and outcomes at the comparator sites that might contribute to residual confounding.

## Conclusions

In conclusion, we found that the implementation of an SQ insulin-driven protocol for treating DKA was associated with substantial changes in practice, including a significantly decreased need for intensive care and reduced rates of readmission, with no increase in hypoglycemia or mortality. Our results suggest that an SQ insulin-driven protocol including the selection of optimal patients can be used effectively in treating DKA, although these results should be confirmed in future prospective studies.

## Notes

Supplement. **eTable.** Missingness of Laboratory Values Data in Diabetic Ketoacidosis Cohort
