# Supplementary material for: Evaluation of Outcomes Following Hospital-Wide Implementation of a Subcutaneous Insulin Protocol for Diabetic Ketoacidosis
Source: JAMA Netw Open. 2022 Apr 7;5(4):e226417. doi: 10.1001/jamanetworkopen.2022.6417 (PMC8990349; doi:10.1001/jamanetworkopen.2022.6417)
Supplement: Supplement. — eTable. Missingness of Laboratory Values Data in Diabetic Ketoacidosis Cohort [file jamanetwopen-e226417-s001.pdf]

Supplemental Online Content

Rao P, Jiang SF, Kipnis P, et al. Evaluation of outcomes following hospital-wide implementation of a subcutaneous insulin protocol for diabetic ketoacidosis. *JAMA Netw Open*. 2022;5(4):e226417. doi:10.1001/jamanetworkopen.2022.6417

eTable. Missingness of Laboratory Values Data in Diabetic Ketoacidosis Cohort

This supplemental material has been provided by the authors to give readers additional information about their work.

© 2022 Rao P et al. *JAMA Network Open.*

eTable. Missingness of Laboratory Values Data in Diabetic Ketoacidosis Cohort

	Intervention Site		Standard Care Site	
	**Pre**	**Post**	**Pre**	**Post**
Hospital encounters	*n = 298*	*n = 122*	*n = 4,441*	*n = 3,128*
Unique patients	*n = 173*	*n = 87*	*n = 2,703*	*n = 2,083*
Glucose	0(0.0)	0(0.0)	0(0.0)	0(0.0)
Anion gap	0(0.0)	0(0.0)	1(0.02)	0(0.0)
Blood urea nitrogen	0(0.0)	0(0.0)	2(0.05)	0(0.0)
Bicarbonate	0(0.0)	0(0.0)	2(0.05)	0(0.0)
Sodium	0(0.0)	0(0.0)	0(0.0)	0(0.0)
White blood cell count	0(0.0)	0(0.0)	9(0.2)	1(0.03)
Creatinine	0(0.0)	0(0.0)	0(0.0)	0(0.0)
Potassium	1(0.3)	0(0.0)	175(3.9)	95(3.0)
Chloride	0(0.0)	0(0.0)	6(0.1)	6(0.2)
Arterial PH	130(43.6)	96(78.6)	1854(41.7)	2376(76.0)
Venous PH	194(65.1)	53(43.4)	2778(62.6)	1132(36.2)

© 2022 Rao P et al. *JAMA Network Open.*
